# Drivers of within-field spatial and temporal variability of crop yield across the US Midwest

**DOI:** 10.1038/s41598-018-32779-3

**Published:** 2018-10-04

**Authors:** Bernardo Maestrini, Bruno Basso

**Affiliations:** Department of Earth and Environmental Sciences, Michigan State University, East Lansing, USA

## Abstract

Not all areas of a farmer’s field are equal; some always produce more relative to the rest of the field, others always less, while still other areas fluctuate in their production capacity from one year to the next, depending on the interaction between climate, soil, topography and management. Understanding why the yield in certain portions of a field has a high variability over time—we call these areas *unstable*—is of paramount importance both from an economic and an environmental point of view, as it is through the better management of these areas that we can improve yields or reduce input costs and environmental impact. In this research, we analyzed data from 338 fields cultivated with maize, soybean, wheat and cotton in the US Midwest to understand how topographic attributes and rain affect yield stability over time. In addition to this high resolution yield monitor dataset, we used publicly available data on topography, rain and soil information to test the hypothesis that within-field areas characterized by a low topographic wetness index (proxy for areas with probability of lower water content) always perform poorly (low and stable yield) compared to the rest of the field because they are drier, and that areas of a field characterized by a mid-high wetness index (high and stable yield) always perform well relative to rest of the field because they have greater water availability to plants. The relative performance of areas of a field with a very high wetness index (e.g. depressions) strongly depends on rain patterns because they may be waterlogged in wet years, yielding less than the rest of the field, or wetter during dry years, yielding more than the rest of the field. We present three different observations from this dataset to support our hypothesis. First, we show that the average topographic wetness index in the different stability zones is lower in low and stable yield areas, high in high and stable yield areas and even higher in unstable yield areas (p < 0.05). Second, we show that in dry years (low precipitation at plant emergence or in July), unstable zones perform relatively better compared to the rest of the field. Third, we show that temporal yield variability is positively correlated (p < 0.05) with the probability of observing gleying processes associated with waterlogging for part of the year. These findings shed light on mechanisms underlying temporal variability of yield and can help guide management solutions to increase profit and improve environmental quality.

## Introduction

Precision Agriculture technologies have the ability to potentially increase yield or reduce environmental impact and input costs through a variable-rate input application^1,2^. This simply translates to what is often defined as the 4 R strategy: the Right thing, at the Right place, at the Right time and in the Right manner. The prescription of input for areas whose yield is consistently higher or lower than the field average is relatively simple; input application can be proportional to the yield, or it can follow recommendation rates based on a strategic management where the input response is known due to previous observations (low N response to low yield areas, or high N response to high yield areas^3,4^). However, the management of areas where yield fluctuates from year to year necessitates a tactical approach^3–5^ based on in-season observations from remote sensing data, or process-based modeling, to determine the crop conditions before a management strategy (e.g. side-dress N application) is implemented.

Previous studies on the effects of topography on crop yield or biomass concluded that yield varies according to position on the landscape^6,7^, but they were limited to small transects or single fields^8,9^ and/or a limited number of seasons^10^.

In this study, we investigated the spatial and temporal variability of crop yield using a large dataset of high-resolution yield monitor data (338 fields, with a minimum of three years of data for each field). Of the 338 fields included in the study, 118 are located in Arkansas, Kansas and Colorado, where irrigation is predominant. The remaining 220 fields are located in states where annual crops are usually rainfed (Michigan, Illinois, Iowa and Indiana). We split the analysis between irrigated and rainfed fields.

The first objective of this study was to compare the magnitude of spatial and temporal variability, where spatial variability represents variations in yield observed within a field in a single year and where temporal variability is the variation in yield observed for each field across the years. This clarifies the relative contributions to yield fluctuations of climate variability (temporal variability), topography and/or soil variability (within-field spatial variability).

Our second objective was to use our yield monitor data to provide evidence for the hypothesis that the interaction between topography and weather is one of the drivers of yield variability from one growing season to the next. The relationship between yield and topographic features such as elevation, curvature, cumulative flow and slope has been previously explored by a multitude of smaller studies that confirm the theory that upper portions of the fields are characterized by lower yields^6,11^. Here we provide evidence for the interaction between weather and topography and hypothesize that this theory explains the mechanisms governing temporal variability of yield. We hypothesize that the depressional areas of a field have a higher temporal variability of yield because they are most likely to be partially waterlogged in wet years (resulting in decreased emergence rate and lower yields) and most likely to be wetter in dry years, when water is a limiting factor during grain filling (resulting in higher yields). In fact, topography is the main driver of waterlogging in the absence of tile drains, as it controls both vertical and horizontal water distribution^12^, as influenced by precipitation patterns.

A similar hypothesis has been previously proposed and validated by Kravchenko and Bullok^6^ in a study of eight corn and soybean fields located in Indiana and Illinois. Kravchenko and Bullok^6^ observed that areas characterized by moderate curvature showed no consistent relation between yield and precipitation. However, they concluded that yield from concave areas was negatively correlated with May precipitation (seedling emergence) and positively correlated with August and September precipitation (grain filling). Kumhálová *et al*.^9^ found in a four-year field experiment in the Czech Republic cropped with rape and cereals that the yield was more strongly correlated with flow accumulation in dry years than in wet years.

Our hypothesis was designed to understand the causes of unstable zones, which we address with evidence from a large dataset.

To facilitate the visualization and interpretation of our observations, we divided each field into three stability classes based on the average productivity and variability across the years of the yield map pixel of the field. The classes were low and stable; high and stable; and unstable as defined in previous studies^4,13,14^.

We provide the following observations to support our hypothesis:

1. Portions of fields located in different stability classes (low yield and stable; high yield and stable; unstable yield) have different topographic wetness index means. The stability class of each cell is based on the average and the standard deviation of observed yield across time.
2. The relative yield performance of unstable zones is negatively correlated to precipitation during plant emergence and grain setting and filling period (for maize vegetative stages, V6 for six leaves fully extended, VT for maize plant with tassel).
3. A positive correlation exists between yield temporal stability and the probability of observing gleying layers. In fact, gleying processes (redistribution of reduced iron along the profile) are indicators that soil is waterlogged, at least for part of the year^15^.
4. The observations 1, 2 and 3 do not hold for irrigated fields because these fields are usually flat and, therefore, the topographic differences play a minor role, and because the irrigation provides the necessary water for the plants without any waterlogging.

## Materials and Methods

### Dataset formation

#### Yield dataset

We analyzed a dataset of about 600 fields with yield maps collected directly from farmers in the US Midwest. We eliminated those fields where less than three years of yield was available and any yield maps where the yield was recorded on less than 75% of the field area. The resulting dataset is comprised of 338 fields and 1625 yield maps (Table S1). Crops grown in these fields included, in order of frequency, maize (*Zea mays L*.), soybean (*Glycine max, L*.), cotton (*Gossypium spp L*.) and wheat (*Triticum spp L.)* as shown in Table S1. Fields located in Arkansas, Colorado and Kansas were identified as irrigated, and fields located in Illinois, Indian, Iowa and Michigan (Figure S1) were identified as rainfed.

We obtained spatial yield data points from datasets collected by yield monitor sensors mounted on farmers’ combines. We transformed georeferenced dry yield data into raster data with a resolution equal to the resolution of the 1 arcsecond (30 m) National Elevation Dataset (NED) digital elevation model (DEM).

Points within a 20-m buffer of the field border were set as missing values to remove the effect of the fill and finish mode error that occurs during harvest monitoring^16^. We also removed the points that were 0.1 times below the median and points that were 3 times above the median of the yield map.

For each field we obtained a digital elevation model from the 1 arcsecond National Elevation Dataset^17^. The average difference between maximum and minimum elevation was 8 m for rainfed fields and 2 m for irrigated fields (Figure S2).

#### Digital elevation model

We used the DEM to calculate the topographic wetness index of each raster cell using the following formula^18^:

$$Topographic\,index=\,\mathrm{ln}(\frac{contributing\,area}{\tan \,slope})$$

where area was measured in m^2^, and slope in radians. We calculated slope over an area corresponding to the field boundary extended by 200 m, to ensure the measurability of the slope at the border of the field, whereas the contributing area was calculated considering null the contributions from the area outside the field.

#### Rain data and planting dates

Twelve years of precipitation data was obtained for each field using the Daymet gridded estimates of daily weather parameters^19^. Because sowing dates for each field were unknown, we used USDA progress crop reports^20^ as a means to estimate those dates. We used USDA weekly crop reports to determine the week of the year when 50% of the land cultivated with the crop of interest had been planted. These values were used as the planting week for each state-year-crop combination. As a proxy of the amount of rain received for the plant to emerge, we used the cumulative rain between one week before the planting week and two weeks after the planting week. We report the planting weeks for maize and soybean in the Supplementary Materials.

#### Gleying

Gleying is defined as the processes of waterlogging in poorly drained soils. We used the Soil Survey Geographic Database^21^ to identify where gleying processes are observable. The SSURGO dataset is spatially explicit and maps soil characteristics with polygons smaller than the fields in our dataset. Fields in the rainfed states in our study averaged 5 polygons for every field, and those in irrigated states averaged 2.5 polygons per field. We used the following algorithm to extract information about the presence of gleying processes from each polygon:

1. We extracted all the map units intersecting a given field.
2. Each map unit is composed of multiple components; the components are not mapped, but an estimate of the (expressed as percentage of the map unit area) of the area occupied by each component is provided. From each map unit we extracted the most representative component using the field *comppct.r*.
3. For each map unit we determined whether any of the soil horizons and layers were designated with the suffix *g* that indicates strong gleying. We searched for the g suffix in the horizon name (field hzname). If strong gleying processes were present, the map unit was marked as TRUE, therefore transforming gleying processes into a Boolean variable.

#### Temporal variability and stability classes

We estimated temporal variability by calculating the standard deviation across the years of the normalized yield. We normalized the yield of every field-year yield map by centering it on 0 and scaling it to a standard deviation of 1, and then for every pixel of every field we calculated the standard deviation of the normalized yield across all the years available for that field.

The division of each field into stability zones and the attribution of a stability class to each raster cell were completed with the following algorithm:

1. We normalized the yield of each field-year yield map as described above.
2. We calculated the temporal variability map for each field as the standard deviation across the years for each cell of the raster. Similarly, we calculated the average normalized yield as the average across the years for each cell of the raster. Cells with at least one missing value were excluded from the computation of the average normalized yield and were categorized as not available.
3. Cells were classified as unstable if their temporal variability was greater than 1 and as stable otherwise.
4. Stable points with an average normalized yield greater than 0 were classified as high and stable. Stable points with an average normalized yield lower than 0 were classified as low and stable.

### Quantification of spatial and temporal variability

We compared spatial and temporal variability separately for each field and crop. We quantified spatial variability as the standard deviation of the distribution of yield observed in each yield map, whereas for the temporal variability we used as an estimator the standard deviation of the averages across the years. We tested for each crop if the difference between temporal and spatial variability differed significantly from 0 by using the Wilcoxon signed ranked test.

### Statistics that support the influence of topography on yield stability

#### Topographic wetness index and yield stability class

We checked the statistical significance of the observation 1 by fitting the following linear mixed effect model to the data. The model was fit separately to the cells in the “irrigated” and “rainfed” state*s*:

$$Topographic\,wetness\,index={\alpha }_{stabilityclass}+{\varepsilon }_{field}+{\varepsilon }_{field-stability}+{\varepsilon }_{residuals}$$

where *α*_*stability class*_ are parameters depending on the stability class estimated in the stability map (low and stable; high and stable; unstable); ${\varepsilon }_{field}$ is a random effect whose levels are the individual fields; ${\varepsilon }_{field-stability}$ is a random effect where the levels are all the possible combinations of field and stability zones; and ${\varepsilon }_{residuals}$ are the model residuals. We tested the differences between the three levels of the parameter *α*_*stability class*_, and applied the Bonferroni correction to the p-value since multiple comparisons were completed for this statistical analysis. Because we compared three stability classes (low and stable; high and stable; and unstable), we had three post-hoc hypotheses. Therefore, the corrected p-value to reject the null hypothesis was 0.05/3 = 0.017.

#### Effect of rain on relative yield performance depending on the stability class

We modeled the relative yield performance of maize and soybean in rainfed states to test the statistical significance of observation 2 using the following linear mixed effect model:

$$logit(yiel{d}_{ranked})={\alpha }_{stabilityclass}+(rain+{\varepsilon }_{field-stability})\ast {\beta }_{stabilityclass}+{\varepsilon }_{residual}$$

where $yiel{d}_{ranked}$ indicates the yield percentiles obtained by each cell relative to the field-year yield map (e.g., for field number 234, year 2015 the pixels were transformed to percentiles so that the lowest would be 0 and the highest 100). Since the percentiles are bounded between 0 and 100, we divided the percentile values by 100 and applied the logit function to expand their domain from [0, 1] to the set of the real numbers (−∞, +∞). We back transformed the results using the inverse logit function. The *α*_*stability class*_ and *β*_*stability class*_ are respectively intercepts and slopes, depending on the stability class estimated in the stability map (low and stable; high and stable; unstable);${\varepsilon }_{field-stability}$ is a random effect of the slope, having as levels the combinations of fields and stability zones; ${\varepsilon }_{residual}$ are the residuals of the model. We fit the model separately using as rain predictor, first the cumulative rain at emergence (see paragraph *Rain data and planting dates* for further details on the determination of the emergence period) and then the cumulative rain in the month of July. To remove any influence of the response variable ($yiel{d}_{ranked}$) on the stability class (one of the predictors) for each year, we calculated the stability map by removing the year. For example, if for a given field the years 2014, 2015, 2016, 2017 were available, the stability map for 2015 was calculated using data for years 2014, 2016 and 2017; while for 2017, data for years 2014, 2015 and 2016 were used.

Our null hypothesis—that unstable and stable portions of a field respond equally to rain during emergence and growing season—is considered falsified if the probability that the model parameters *β*_*stability class*_ are equal is lower than 0.05.

#### Presence of gleying processes as determinant of yield temporal variability

We tested whether yield temporal variability may explain the presence of gleying processes using the following model, fit separately to the subset of fields in irrigated states and in rainfed states:

$$gleying=\alpha +temporal\,variability\ast \beta +{\varepsilon }_{county}+{\varepsilon }_{residuals}$$

where *α* and *β* are the slope and intercept parameters; temporal variability is the average of the temporal variability measured within each map unit polygon; ${\varepsilon }_{county}$ is a random effect of the intercept whose levels are the counties where our fields are located; and ${\varepsilon }_{residuals}$ are the model residuals. For this model, the single observations were the map units of SSURGO; for each map unit we averaged the temporal variability observed at its interior; therefore, the total number of observations was the number of map units intersecting our fields.

We used R (version 3.2) extended by the packages raster^22^ for the operations on the spatial data and by the packages lme4^23^ for the linear mixed models.

## Results

**Figure 1:**
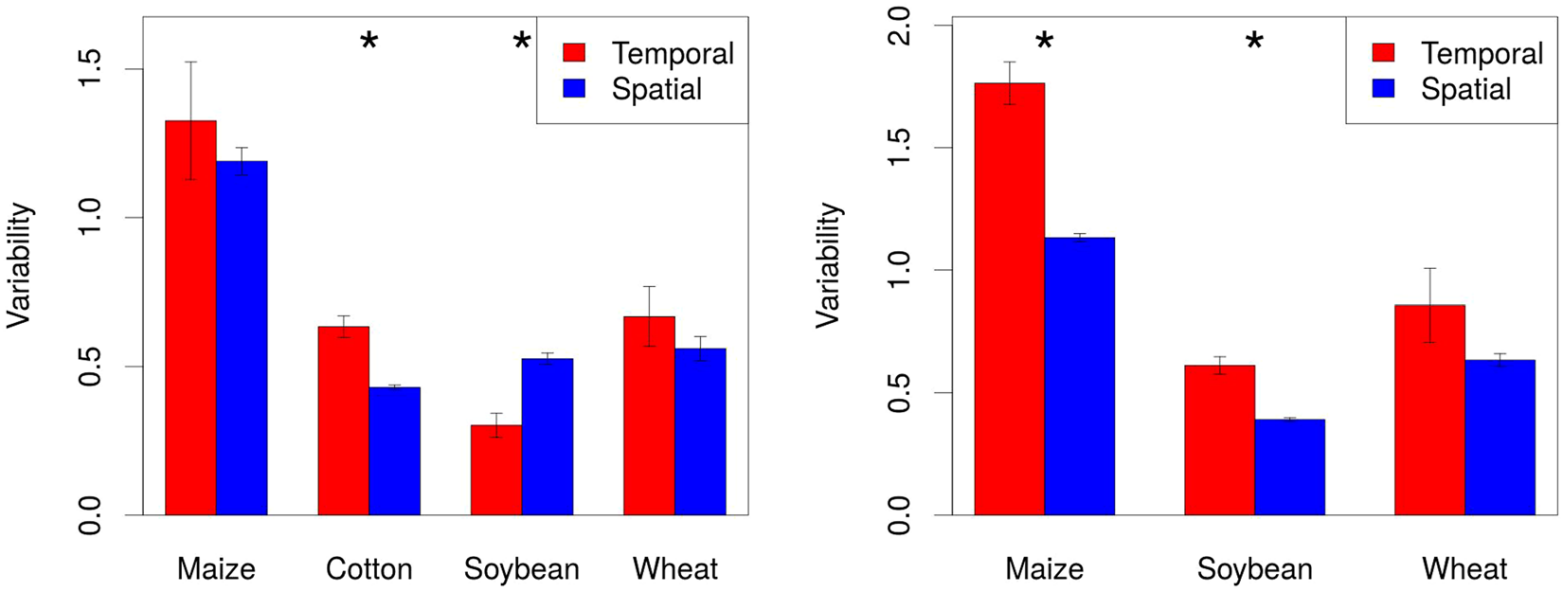
Temporal and spatial variability. Comparison of temporal and spatial variability by crop.

We found that the temporal variability was larger than the spatial within-field variability for every crop in all fields (Fig. 1). Statistical analysis confirmed the statistical significance of our three observations.

Using our classification algorithm, approximately 50% of the field was categorized as high and stable; 32% as low and stable; and 18% as unstable (Table S2).

**Figure 2:**
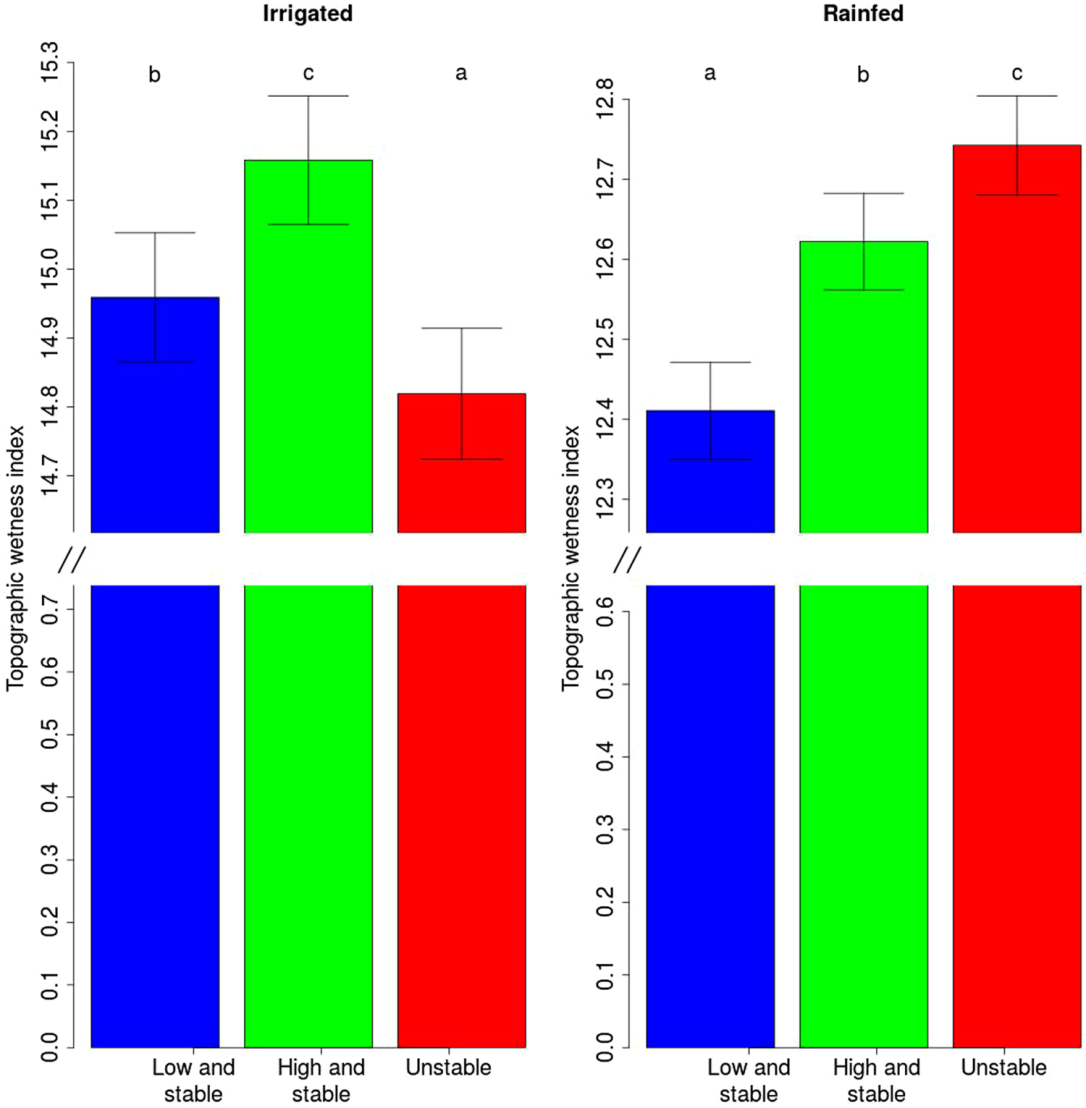
Topographic wetness index of the different stability zones of the field. The bars are the standard error of the fixed effect term in the model explaining the topographic wetness index as a function of the stability level (i.e. the standard error of the parameters *α*_*stability class*_) and the letters indicate significant differences between the averages.

The portions of rainfed fields classified as unstable by our algorithm had, on average, a higher topographic wetness index (12.9) when compared to both the low stable and high stable portions of the field (respectively, 12.4 and 12.7, p < 0.05, Fig. 2). This was not true for irrigated fields, where the unstable portions of the field were associated with the lowest topographic indices (Fig. 2). We repeated this analysis using only one crop at a time to produce the stability maps in fields where the same crop was grown for more than one year. In this case similar trends were observed, but no significant difference was observed between the average topographic index of the high and stable and the topographic index of the unstable portions of the field (Figure S7).

**Figure 3:**
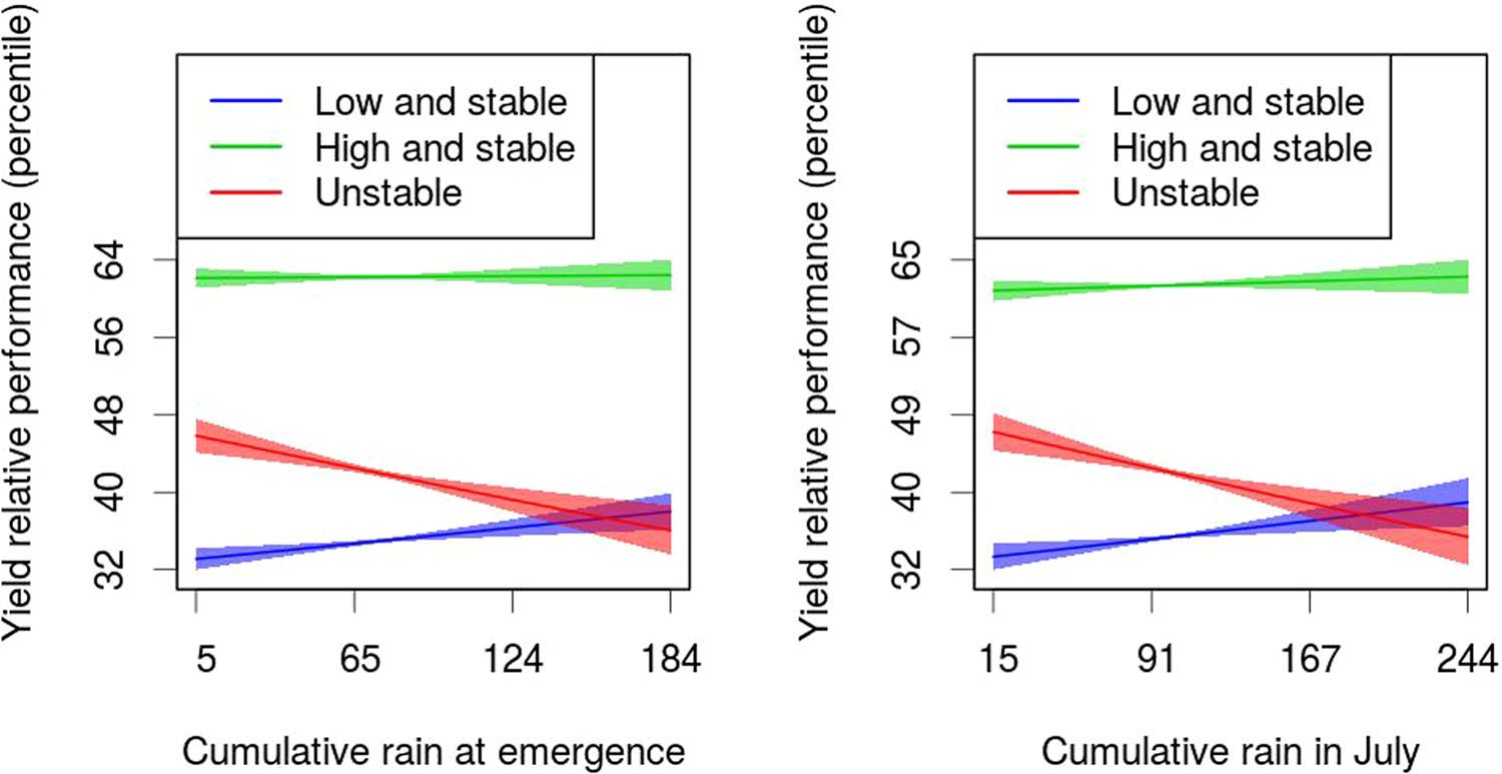
Yield relative performance as a function of stability class and cumulative rain. The figure shows the predictions of the relative yield the predictions have been back-transformed using the inverse logit function. The colored areas indicate the 95% confidence interval of the prediction based solely on the standard error of the fixed effects.

Second, the relative performance of unstable zones was negatively correlated with both the cumulative rain at emergence and cumulative rain in July (Fig. 3, p < 0.05), whereas the relative performance of high and stable zones was substantially unaffected by the amount of rain received. In addition, unstable zones were positively correlated with cumulative rain (Fig. 2).

**Figure 4:**
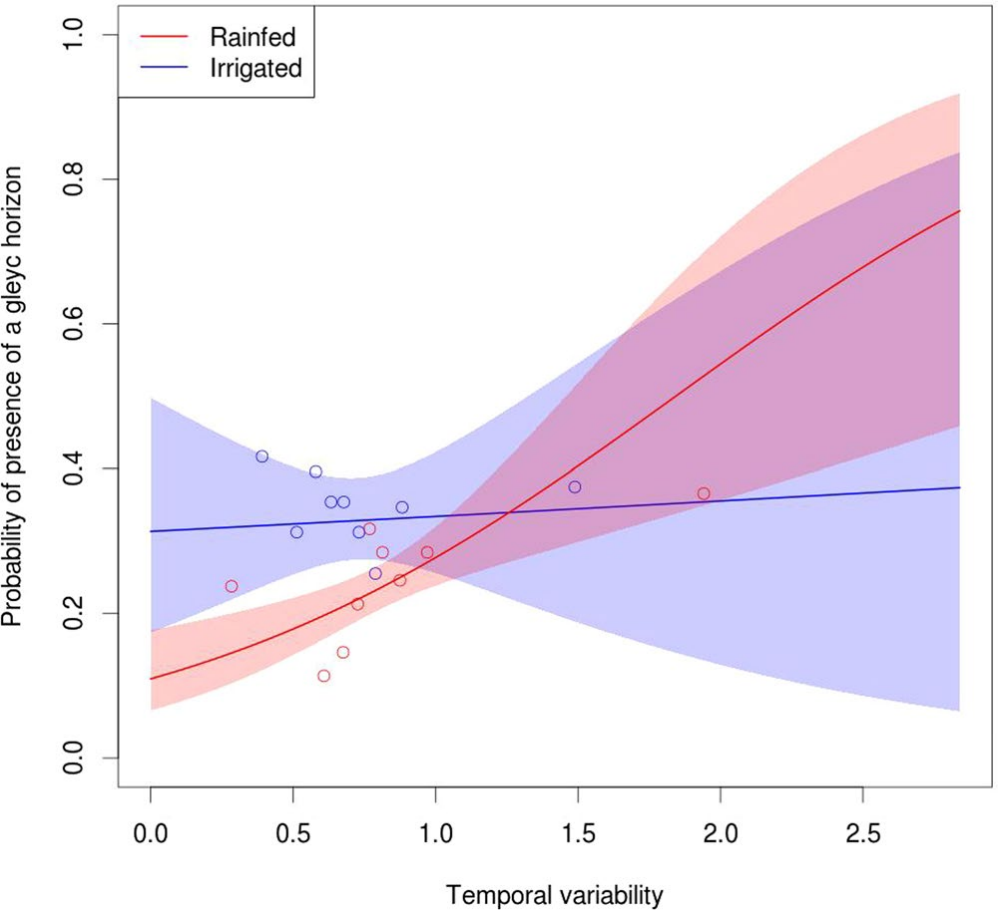
Correlation between the probability of having a gleyc horizon (data binned in deciles) and yield temporal variability. The colored areas indicate the 95% confidence interval of the prediction based solely on the standard error of the fixed effects.

**Figure 5:**
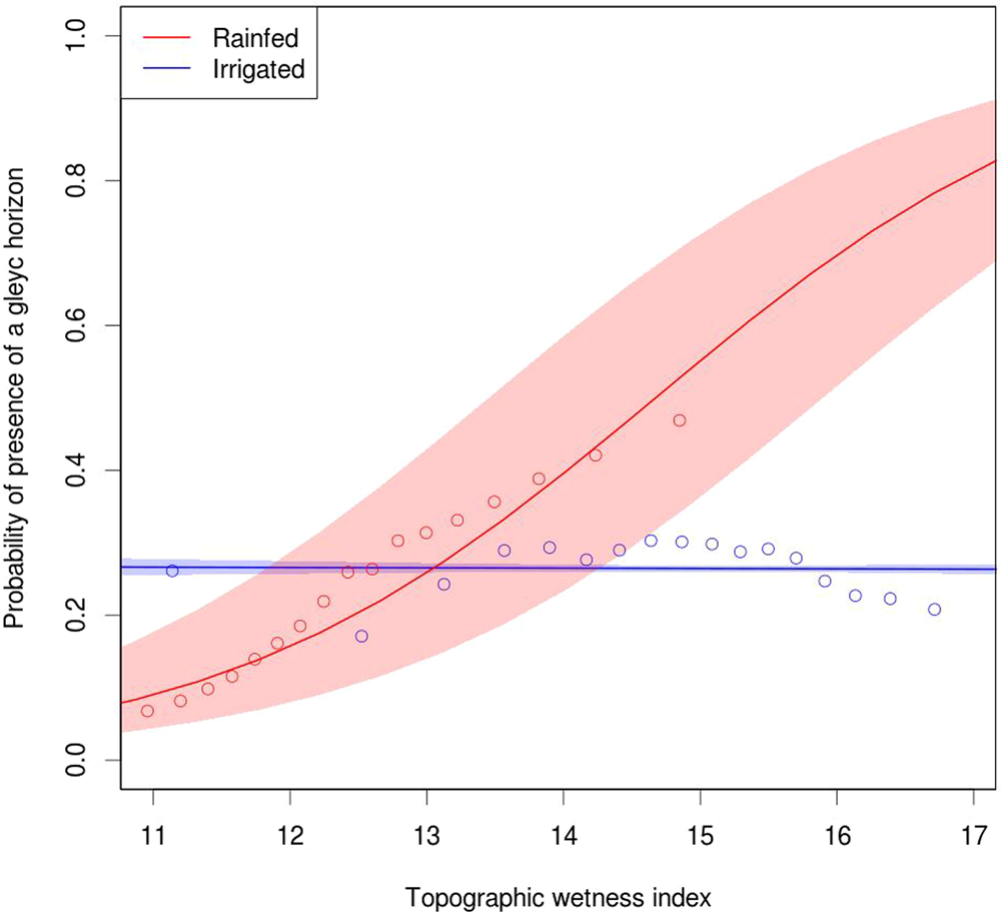
Probability of gleying processes as a function of the topographic wetness index. The line shows the probability of having gleying processes as a function of the topographic index, the data have been binned in 17 quantiles. The colored areas indicate the 95% confidence interval of the prediction based solely on the standard error of the fixed effects.

Our third observation supporting the hypothesis that yield stability is topography-driven is that the probability of observing gleying processes increases with yield temporal variability in fields located in rainfed states (Fig. 4, p < 0.05), whereas this trend was not observed for fields in irrigated states (Fig. 4). As expected, we observed a positive correlation between the topographic wetness index and the probability of observing gleying processes (Fig. 5, p < 0.05).

## Discussion

### Yield temporal and spatial variance

Temporal variance was generally larger than spatial variance. This has important implications for field management and for precision agriculture. In fact, precision agriculture prescription maps with the application of variable rate input are often based solely on soil maps, neglecting interactions between soil, weather and management that may result in vastly different yields in different years. Simply put, this implies that if yield variability was only driven by the spatial variability of soils, yield maps would always look alike, yet they seldom do when compared over the years. The concept of a stability map with a detailed understanding of why some areas of the field always produce more than others and of areas that change over the years, with some years giving high yield and other years giving low yield, is extremely important and rather novel when presented with the type of analysis we report in this paper or in Basso *et al*.^14^ and Maestrini and Basso^4^. Understanding unstable zones is important for in-season adaptive management, better defined as tactical management^3^, to adapt to the deviation from original strategic approach (before the season management plan) as a result of a unique set of weather conditions. In-season observation to understand whether an unstable zone is behaving like a high yielding zone or like a low yielding one in a specific year provides the farmer with a useful insight to decide whether or not to apply sidedress Nitrogen (N) fertilizer to that area. If the year has been favorable, this kind of zone should be managed just like the high yielding zones, but if the weather did not cooperate, then it should be managed like the low yielding zones. For example, crop simulation modeling with a simulated response function of N fertilization rates is critical for determining the amount of fertilizer to be applied based on the condition of the crops till the time of side-dressing and on different forecast scenarios for the remaining of the season. This approach allows farmers to reduce risks when making decisions, and it also provides them with an idea of the optimal N fertilizer as tradeoff between profit and environmental impact.

### The interaction of topography and rain determines yield at within-field scale

Our study confirmed the theory that zones with topographic attributes that allow for the right water accumulation (neither much runoff, nor waterlogging) are associated with higher yields. This theory has been proposed in different formulations, using topographic index^24^, relative elevation^25^, landscape position^26,27^ and curvature^28,29^ as a proxy for topography. Our study provided an extensive validation of this theory and showed that this principle holds true in the stable portions of the field.

Using three independent observations derived by matching our yield dataset with three independent and publicly available datasets, we determined that the interaction between topography and weather is a driver of yield stability.

Our observations support the thesis that the relative performance of the unstable zones is good when a dry period occurs and poor when precipitation is excessive. To investigate the potential impact of different rain patterns, we created a map of the correlation between the cumulative rain in May and July using the data recorded by 571 weather stations in the Midwest over the last century (Figure S6). The map showed that this correlation was generally low, ranging between 0 and 0.17, although some individual weather stations exhibited a higher positive or negative correlation (min correlation −0.56, max correlation 0.7). The map showed no correlation in areas more heavily influenced by the Great Lakes (r coefficient ca. 0, Michigan, Illinois, Indiana, Iowa), with slight increments in the states farther away from the Great Lakes. The lack of correlation between precipitation in May and July shows that having both an extremely wet (or dry) emergence and filling period is unlikely.

We can interpret the influence of rain patterns in the unstable zones in light of their topographic and hydrological characteristics. To support the assumptions behind our hypothesis—that unstable zones are located in depressions and are more prone to waterlogging—we showed that unstable zones have a higher topographic wetness index (Fig. 2), that a correlation exists between topography and yield temporal variability, and that the probability of observing a soil horizon subject to gleying processes is positively correlated to yield temporal variability.

Our model explained yield temporal variability as a function of field-scale topography and rain in rainfed areas. As far as irrigated areas are concerned, fields are usually flatter; therefore differences in topography are usually less pronounced. In fact, irrigated fields generally exhibit a higher topographic wetness index (Fig. 3) and a lower elevation range (Table S2).

Understanding the underlying causes of yield temporal variability has important practical consequences. For example, if attaining the yield potential in an unstable area is hindered by low emergence rates due to waterlogging, that portion of the field may benefit from tile drains. Another important implication of our findings is that areas that can benefit more from tiling may not be those characterized by overall low yields but may instead be those that exhibit larger temporal variabilities in yield.

We stress here the importance of having a sufficient number of years to produce the stability maps. In fact, when the stability maps were produced using only one crop (Figure S7), we were not able to detect significant differences between the average topographic wetness index of the high and stable and the unstable portions. This could be due to a decrease in the statistical power of our analysis (fewer fields had at least two years with the same crop) or a decrease in the precision of the reliability of the stability maps. In fact, a lower number of available years reduces the accuracy in the estimate of the average and the standard deviation of the normalized yield.

## Conclusion

Using a large yield dataset and independent observations, we showed that the interaction between field-scale topography and rain patterns is an important driver of yield, across several common crops of the US Midwest. This occurs because yield variation is highly affected by two hydrological processes: waterlogging in wet springs and grain filling in the summer. Our observations rely on two assumptions: the first being that farmers utilized fertilization rates that were uniform in space and time and the second being that no tile system was in place in any of the fields. Although it is unlikely that all fields met our assumptions, we don’t believe that our findings were significantly weakened. For example, we found a correlation between topography and temporal stability even though some of the fields were certainly tiled and thereby less likely to be waterlogged during emergence. This only strengthens our hypothesis that waterlogging is a cause of yield instability. Because our models are mostly inferential, that is, they serve the purpose of supporting our hypothesis, they have limited predictive power due to the large ecological heterogeneity that exists within our scope of inference. But in conclusion, we showed that the concept of stability zones lead to a different approach, compared to the original idea of precision agriculture, based on farming by soil. Our study introduces a novel insight, which is farming by stability zones, but it relies on the concept of strategic management for the stable zones and tactical management for unstable zones, using within-season observations (remote sensing) of the crops to determine the fate of the unstable zones and then adapting management to circumstances with a tactical management strategy.

## Electronic supplementary material

Supplementary Information

## References

[1] Basso Bruno, Dobrowolski James, McKay Channing (2017). From the Dust Bowl to Drones to Big Data: The Next Revolution in Agriculture. Georgetown Journal of International Affairs.

[2] Basso B (2016). Environmental and economic benefits of variable rate nitrogen fertilization in a nitrate vulnerable zone. Sci. Total Environ..

[3] Basso B, Ritchie JT, Cammarano D, Sartori L (2011). A strategic and tactical management approach to select optimal N fertilizer rates for wheat in a spatially variable field. Eur. J. Agron..

[4] Maestrini B, Basso B (2018). Predicting spatial patterns of within-field crop yield variability. F. Crop. Res..

[5] Dumont B (2015). Systematic analysis of site-specific yield distributions resulting from nitrogen management and climatic variability interactions. Precis. Agric..

[6] Kravchenko AN, Bullock DG (2000). Correlation of corn and soybean grain yield with topography and soil properties. Agron. J..

[7] Kravchenko AN, Robertson GP, Thelen KD, Harwood RR (2005). Management, Topographical, and Weather Effects on Spatial Variability of Crop Grain Yields. Agron. J..

[8] Beehler J, Fry J, Negassa W, Kravchenko A (2017). Impact of cover crop on soil carbon accrual in topographically diverse terrain. J. Soil Water Conserv..

[9] Kumhálová J, Kumhála F, Kroulík M, Matějková Š (2011). The impact of topography on soil properties and yield and the effects of weather conditions. Precis. Agric..

[10] Ladoni M, Basir A, Robertson PG, Kravchenko AN (2016). Agriculture, Ecosystems and Environment Scaling-up: cover crops differentially in fl uence soil carbon in agricultural fi elds with diverse topography. “Agriculture, Ecosyst. Environ..

[11] Iqbal J, Read JJ, Thomasson AJ, Jenkins JN (2005). Relationships between Soil–Landscape and Dryland Cotton Lint Yield. Soil Sci. Soc. Am. J..

[12] Verity GE, Anderson DW (1990). Soil erosion effects on soil quality and yield. Can. J. Soil Sci..

[13] Blackmore S (2000). The interpretation of trends from multiple yield maps. Comput. Electron. Agric..

[14] Basso B, Bertocco M, Sartori L, Martin EC (2007). Analyzing the effects of climate variability on spatial pattern of yield in a maize-wheat-soybean rotation. Eur. J. Agron..

[15] Pipujol MD, Buurman P (1994). The distinction between ground-water gley and surface-water gley phenomena in Tertiary paleosols of the Ebro basin, NE Spain. Palaeogeogr. Palaeoclimatol. Palaeoecol..

[16] Lyle G, Bryan BA, Ostendorf B (2014). Post-processing methods to eliminate erroneous grain yield measurements: Review and directions for future development. Precis. Agric..

[17] USGS. *1 arc-second Digital Elevation Models* (*DEMs*) (2017).

[18] Beven KJ, Kirkby MJ (1979). A physically based, variable contributing area model of basin hydrology. Hydrol. Sci. Bull..

[19] Thornton, P. E. *et al*. *Daymet: Daily Surface Weather Data on a 1-km Grid for North America, Version* **3** (2017).

[20] NASS. Crop Progress. *Crop progress report* (2017). Available at: http://usda.mannlib.cornell.edu/MannUsda/viewDocumentInfo.do?documentID=1048 (Accessed: 1st December 2017).

[21] USDA. Soil Survey Geographic (SSURGO) Database. (2017). Available at: https://sdmdataaccess.sc.egov.usda.gov (Accessed: 1st December 2017).

[22] Hijmans, R. J. raster: Geographic Data Analysis and Modeling (2016).

[23] Bates, D., Maechler, M., Bolker, B. & Walker, S. lme4: Linear mixed-effects models using Eigen and S4 (2015).

[24] Bao Liang C, Cheng SB, Walley F, Yates TT (2009). Indices and Yield Variability in a Rolling Landscape of Western Canada. Pedosphere.

[25] Kaspar TC (2003). Relationship between six years of corn yields and terrain attributes. Precis. Agric..

[26] McConkey BG, Ulrich DJ, Dyck FB (1997). Slope position and subsoiling effects on soil water and spring wheat yield. Can. J. Soil Sci..

[27] Changere A, Lal R (1997). Slope Position and Erosional Effects on Soil Properties and Corn Production on a Miamian Soil in Central Ohio. J. Sustain. Agric..

[28] SINAI G., ZASLAVSKY D., GOLANY P. (1981). THE EFFECT OF SOIL SURFACE CURVATURE ON MOISTURE AND YIELD—BEER SHEBA OBSERVATION. Soil Science.

[29] Simmons FW, Cassel DK, Daniels RB (1989). Landscape and Soil Property Effects on Corn Grain Yield Response to Tillage. Soil Sci. Soc. Am. J..

