# Supplementary material for: Drivers of within-field spatial and temporal variability of crop yield across the US Midwest
Source: Sci Rep. 2018 Oct 4;8:14833. doi: 10.1038/s41598-018-32779-3 (PMC6172268; doi:10.1038/s41598-018-32779-3)
Supplement: Supplementary file 1 — Supplementary Information [file 41598_2018_32779_MOESM1_ESM.docx]

Supplementary Material for:

**Drivers of within-field spatial and temporal variability of crop yield across the US Midwest**

Bernardo Maestrini and Bruno Basso

Department of Earth and Environmental Sciences,

Michigan State University, East Lansing

**Description of the supplementary materials**

The supplementary materials include the map of the location of the fields (Figure S1), the distribution of the elevation ranges within each field (Figure S2), the progress of the number of fields planted over the course of the year estimated by the USDA for each combination of state-year for soy and maize, the distribution of the number of map units mapped by SSURGO for each field (Figure S3), the distribution of the number of years available for each field (Figure S4), the distribution of the size of the fields included in this study (Figure S5), and a Midwest map of the correlation between cumulative rain in May and July. We also report a contingency table showing the number of fields and farmers divided by crop and state included in this study (Table S1), and the percentages of the field occupied by the different stability zones (Table S2).


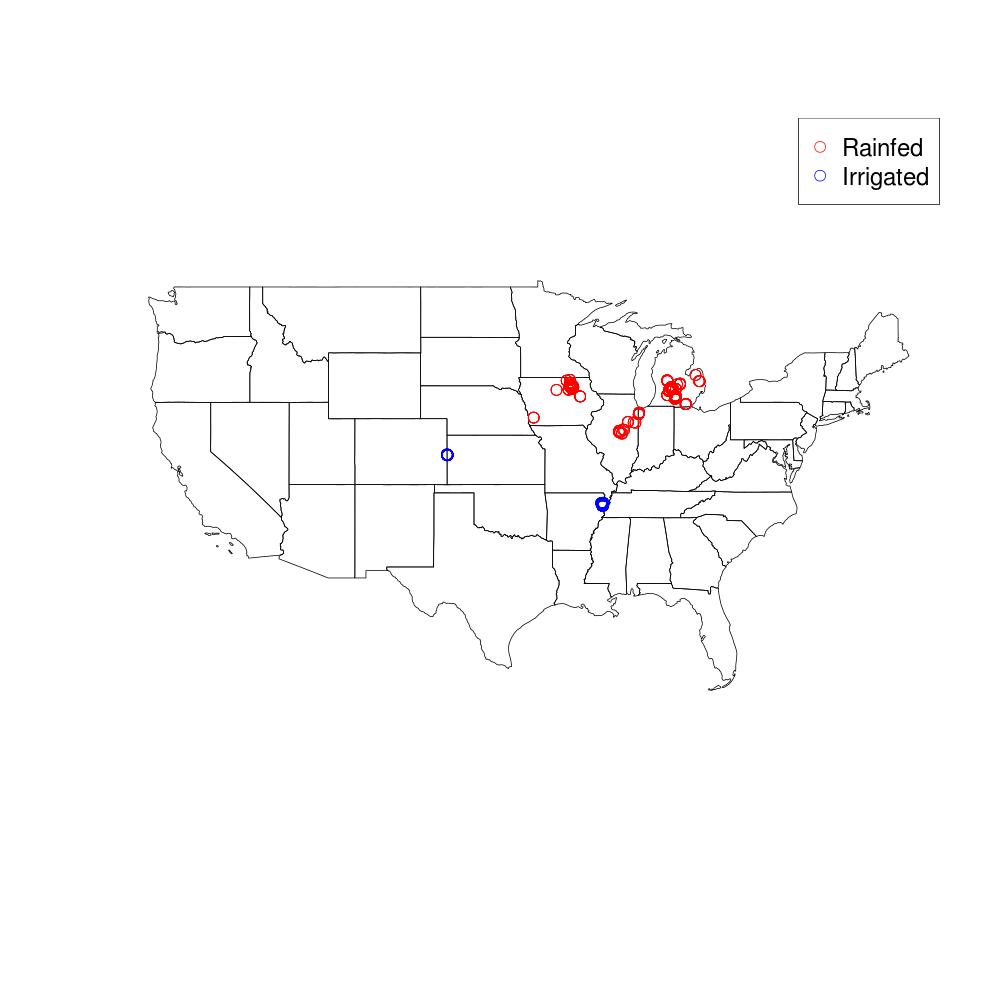

Figure S1: Map of the geographical location of the fields used in this study. The points in red indicate the fields located in states where annual crops are more frequently rainfed, whereas the blue points are the fields located in states where the annual crops are more often irrigated.


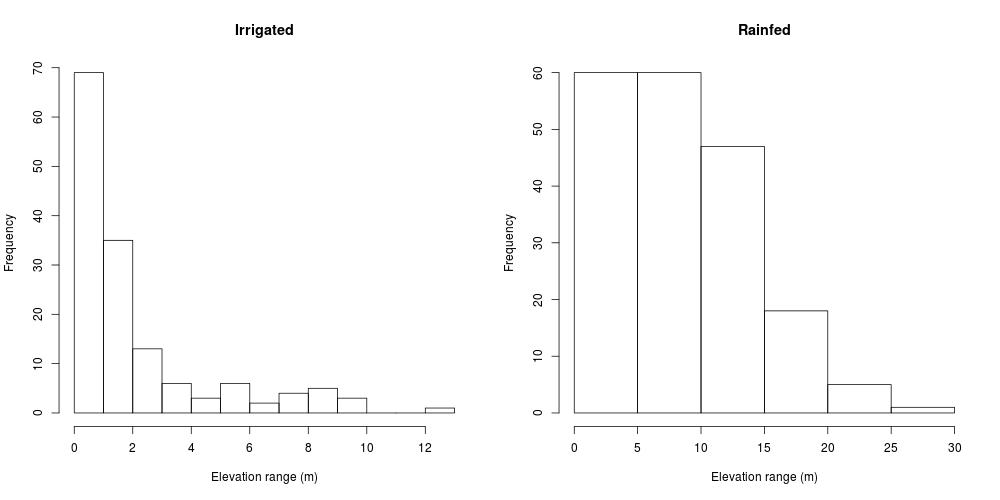


Figure S2: Distribution of range of elevation (i.e. max elevation - min elevation) observed within each field in irrigated and rainfed states


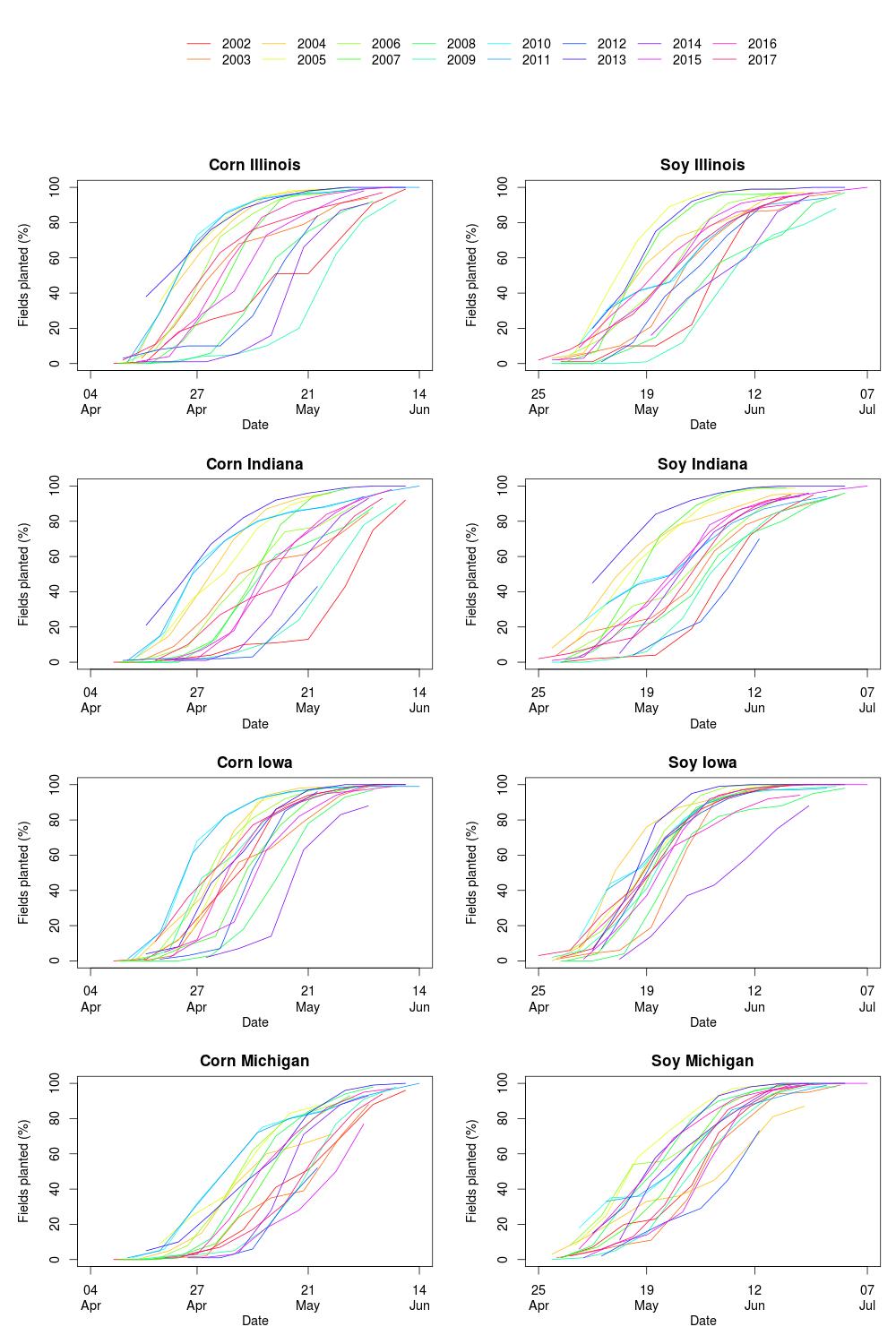


Figure S3: Distribution of the completion of plantation in the field surveyed by the USDA for the crop progress report.


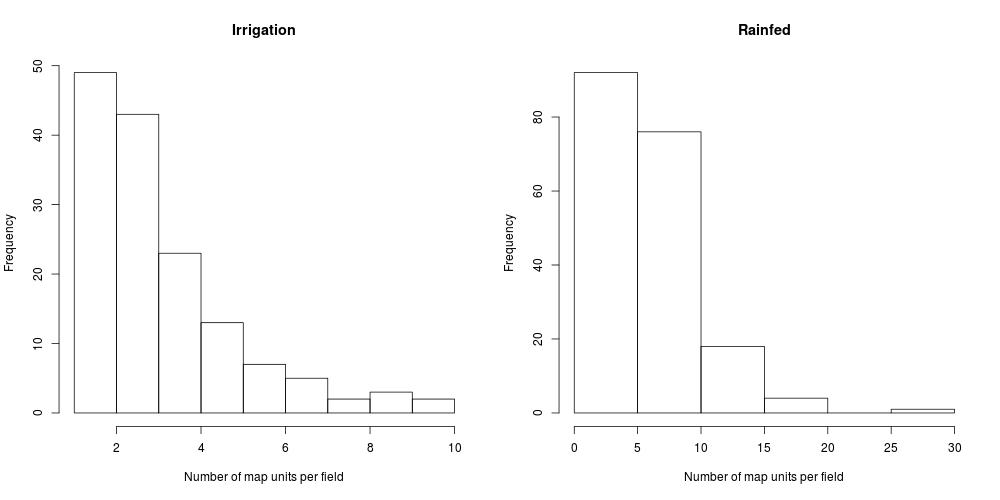

Figure S4: Distribution of the number of map units for each field identified by the Soil Survey Geographic Database.


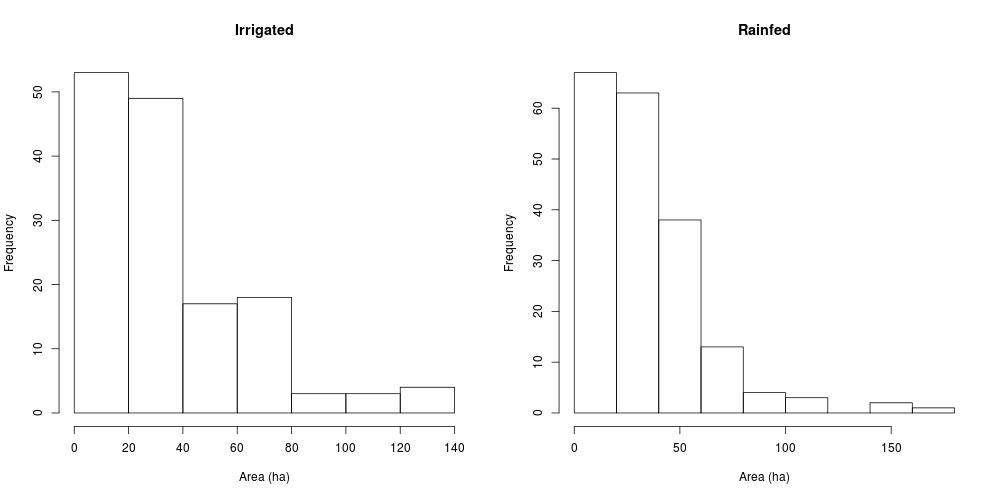

Figure S5: Distribution of the size of the field in irrigated and rainfed states.

*
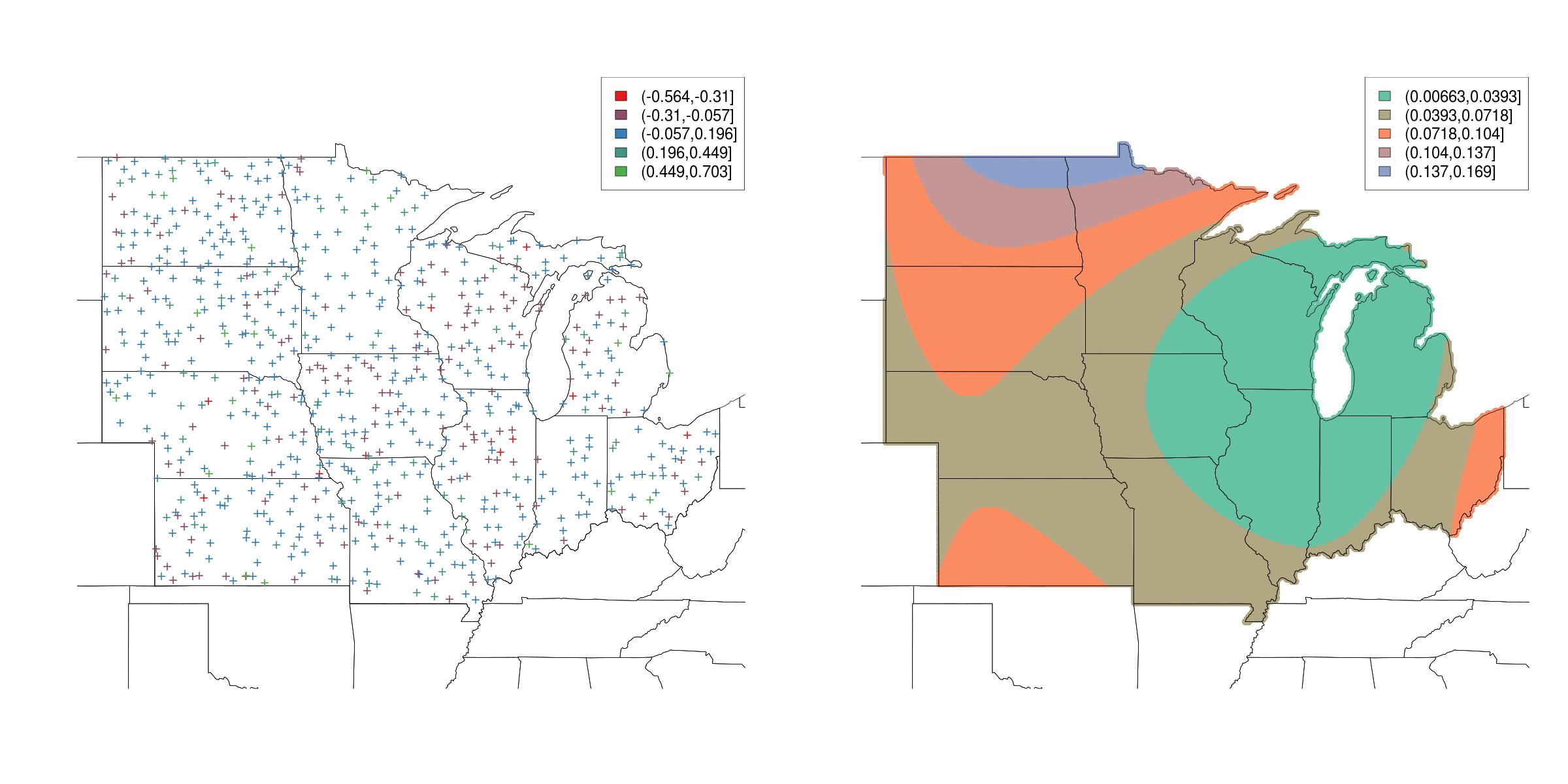
Figure S6: Geographic distribution of the correlation between the cumulative rain in May and in July. The map was produced using the meteorological data from the Global Historical Climatology Network. We downloaded data from 571 meteorological stations having each at last ten years of precipitation recorded. For each station we calculated the correlation between the cumulative rain in May and in July to obtain a set of point (figure on the left). We then used a generalized least square third degree polynomial model to simulate the surface trend of the correlation between the cumulative rain in May and July. To account for spatial correlation between the points we estimated the spatial correlation structure using a linear function.*

Table 1: Contingency table reporting the number of harvest map by state and crop. The number in parenthesis report the number of farmers (each farmer has reported typically more than one field).

|  | Arkansas | Colorado | Illinois | Indiana | Iowa | Kansas | Michigan | Sum |
| --- | --- | --- | --- | --- | --- | --- | --- | --- |
| Maize | 65(2) | 18(1) | 18(8) | 13(1) | 218(30) | 30(1) | 349(16) | 711(59) |
| Cotton | 266(2) | - | - | - | - | - | - | 266(2) |
| Soy | 117(2) | - | 18(7) | 8(1) | 87(29) | - | 314(15) | 544(54) |
| Wheat | - | 8(1) | - | - | - | 25(1) | 71(6) | 104(8) |
| Sum | 448(6) | 26(2) | 36(15) | 21(2) | 305(59) | 55(2) | 734(37) | 1625(123) |
|  |  |  |  |  |  |  |  |  |
| N. of fields | 118 | 7 | 12 | 2 | 57 | 22 | 120 | 338 |
|  |  |  |  |  |  |  |  |  |
| N. of famers | 2 | 1 | 8 | 1 | 30 | 1 | 16 | 59 |

Table 2: Percentages of the field occupied by te stability zones in rainfed and irrigated areas.

|  | High and stable | Low and stable | Unstbale |
| --- | --- | --- | --- |
| Rainfed | 32(6) | 51(8) | 17(9) |
| Irrigated | 33(6) | 49(7) | 19(9) |


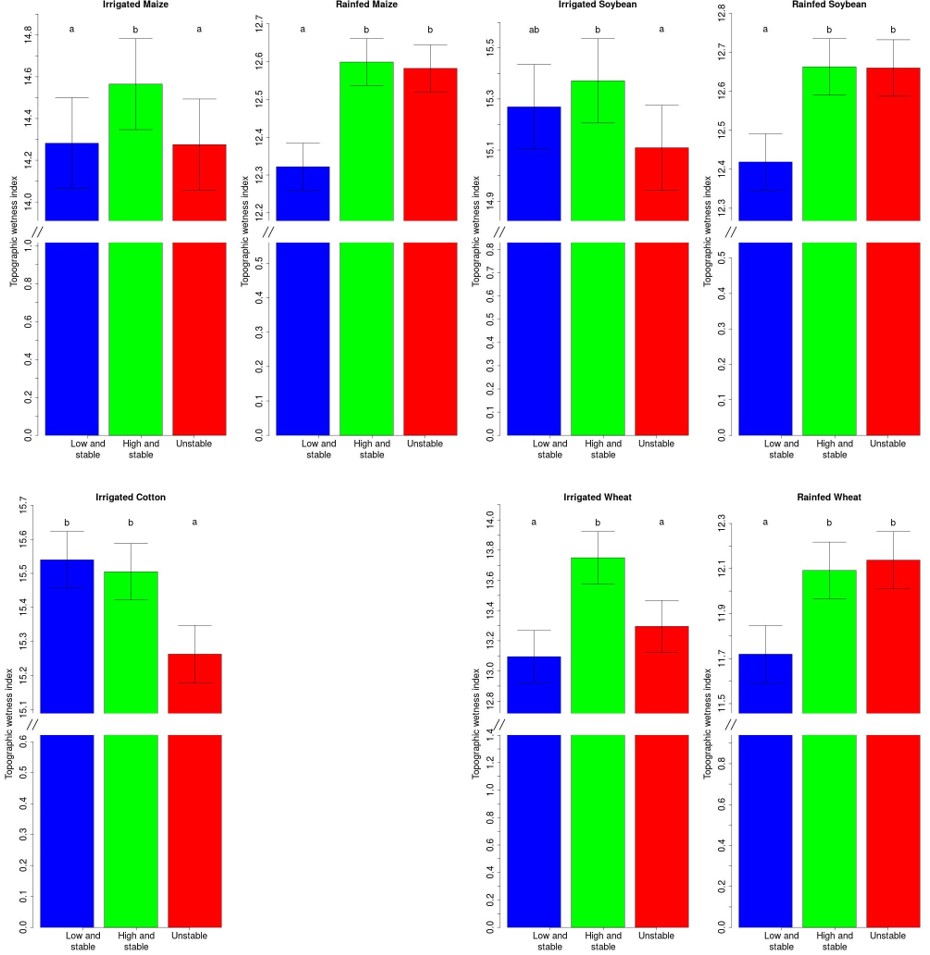


Figure S7: *We repeated the analysis on the correlation between Topographic Wetness Index and the stability classification (Figure 1) by using only one crop at a time to form the stability maps. The stability maps were created using the same algorithm described in the “Temporal variability and stability classes section” but using exclusively data from one crop at a time (i.e. we created the stability maps first using only the years cropped with maize, then only the years cropped with soybean, then cotton and finally wheat). The number of fields available for this analysis was lower because only few fields were available at least two years with the same crop. The number of fields in the analysis is reported in the table here below*.

|  | Maize | Cotton | Soybean | Wheat |
| --- | --- | --- | --- | --- |
| Rainfed | 156 | 0 | 138 | 13 |
| Irrigated | 31 | 74 | 34 | 16 |
